# Optimizing A Lipocomplex-Based Gene Transfer Method into HeLa Cell Line

**Published:** 2013-11-20

**Authors:** Alimohammad Asgharian, Mehdi Banan, Hossein Najmabadi

**Affiliations:** 1 Department of Cell and Molecular Biology, Islamic Azad University, Tonekabon Branch, Tonekabon, Iran; 2 Genetics Research Center, University of Social Welfare and Rehabilitation Sciences, Tehran, Iran

**Keywords:** Transfection, HeLa Cells, Lipids

## Abstract

One of the most significant steps in gene expression studies is transferring genes into cell cultures. Despite there are different methods for gene delivery such as viral and non-viral producers, some cationic lipid reagents have recently developed to transfect into mam- malian cell lines. The main aim of this study was optimizing and improving lipocomplex based transient transfection procedures into HeLa cell line which is being used widely as a typical cell in biological studies.

This study was an experimental research. In this work, pCMV β-Gal DNA plasmid was used as a reporter DNA for determining the rate of gene transfection into HeLa cells. To accomplish the highest gene delivery into HeLa cells, optimizing experiments were carried out in different volumes of FuGENE-HD, Lipofectamine^TM^2000 and X-tremeGENE. Also, we investigated tranasfection efficiency in presence of various cell densities of HeLa cells. Then, transfection efficiency and cell toxicity were measured by beta gal staining and trypan blue methods, respectively.

Using FuGENE-HD in volume of 4µl along with 10^5^ HeLa cells, transfection efficiency was higher (43.66 ± 1.52%) in comparison with the cationic lipids Lipofectamine^TM^2000 and X-tremeGENE. In addition, the rate of cell toxicity in presence of FuGENE-HD was less than 5%.

In summary, the cationic lipid FuGENE-HD indicates a suitable potential to transfer DNA into HeLa cells and it can be an efficient reagent for gene delivery for HeLa cells *in vitro*. Moreover, it is worth designing and optimizing gene transfer experiments for other cell lines with FuGENE-HD due to its low toxicity and high efficiency.

HeLa cell line is a human epithelial cell line that was derived from a patient with cervical carcinoma about 50 years ago (1,2). Indeed, HeLa cell line is one of the most common typical cells as it is being used widely in many research areas such as cancer research, effects of drugs on cells and gene regulation studies (1). There are some reasons for using HeLa cells as a model cell in *in vitro* studies. First of all, HeLa cells are mostly easy to grow and they double through 23 hours (2). Second, obtaining information of different processes that occur in human cells would be possible using HeLa cells (3).

In most studies such as gene knock down and gene cloning, having an appropriate level of gene delivery into cell cultures is necessary (4). There are different gene delivery systems into mammalian cells including viral and non-viral ways but because of variability in efficiency of transfection into various eukaryotic cells, the transfection condition should be optimized for each cell line understudy (4). Thus, transfection efficiency for cell cultures is being taken into consideration before more studies are implemented.

In gene transfer into mammalian cells, viral and non-viral ways each have unique characteristics (4,5). First of all, there are some nonviral gene delivery methods such as electroporation, calcium phosphate precipitation and cationic lipid based transfection with various efficiency and application (4). For instance, in electroporation procedure not only the level of cell toxicity is more comparable with other methods, but also transfection efficiency is not considerable and performing these experiments are mostly too time consuming, because of optimizing electroporation condition including voltage, number of pulse and temperature (6).

On the other hand, calcium phosphate precipitation procedure, which is a non-viral way, is an appropriate way for introducing DNA fragments into some cell lines but it is not efficient for other cell lines (7).

However, non-viral based transfection would be preferred for gene delivery in comparison with viral based gene vectors because of more safety, immunogenicity and simplicity (7-9). Also, since viral carriers might cause integration mutation into genome, it remains a serious concern about using them as gene vectors (10).

In the last few years, some cationic lipids have been developed that are mostly efficient besides other non-viral methods (11,12). Most of cationic lipids are synthetic such as lipofectamine, cellfectin, Highperfect, FuGENE that are produced by various biotechnology companies and each one has different capability for introducing DNA or RNA molecules into cells. To achieve acceptable lipid based transfection some parameters should be optimized such as cell density on the time of transfection and the amount of lipid to DNA (13). In this context, commercially available cationic lipid reagents such as Lipofectamine^TM^2000 (Invitrogen, USA) and X-tremeGENE (Invitrogen, USA) have been highly recommended and referred for cell transfection by their manufacturers, while performing transfection experiments by using them do not always give appropriate results.

Therefore figuring out an efficient gene delivery procedure and determining the best lipid reagent for nucleic acid transfer is absolutely necessary. In this paper, we report an acceptable transfection rate of HeLa cells in presence of FuGENE-HD with pCMV: β-Gal vector as reporter plasmid. To accomplish the highest transfection efficiency, some factors like cell density and lipid amount have been investigated. Afterwards, the capability of some cationic lipid reagents including Lipofectamine^TM^2000 and X-tremeGENE for gene delivery into HeLa adherent cells were analyzed and compared with FuGENE-HD as well.

Our finding pointed that lipid-based gene delivery based on using FuGENE-HD is an appropriate procedure to mediate DNA transfer into HeLa cells because of their lower cytotoxicity and higher transfection in comparison with Lipofectamine^TM^2000 and X-tremeGENE.

This study was an experimental research that was funded by Islamic Azad University and University of Welfare and Rehabilitation Sciences.

HeLa cell line, obtained as a gift from the cell biology lab in Erasmus University, was cultured in Dulbecco’s modiﬁed Eagle’s medium (DMEM) supplemented with 10% fetal calf serum (Biosera, Korea) and 1% penicillinstreptomycin solution (Biosera, Korea) (7). The plasmid pCMV: β-Gal (Promega) encoding the beta-galactosidase protein was used to measure transfection efficiency (8). Subsequently, pCMV: β-Gal was transformed into *Escherichia coli* DH5 for amplification. After that, pCMV: β-Gal plasmids were purified by the plasmid DNA extraction kit (Roche, Switzerland) according to the manufacturer ’s protocol.

In order to maximize transfection efficiency, two parameters were considered including the different volumes of cationic lipid reagent and cells densities. Also amount of plasmid DNA had been kept fix as it was illustrated by kit protocols. Furthermore, because of adverse effects of serum and antibiotics on transfection and cell death, all of transfection experiments were carried out in absence of serum and antibiotics (14-16). Also, transfection experiments were repeated three times independently for each condition as described below.

To accomplish the highest gene delivery by FuGENE-HD, Lipocomlex (FuGENE-HDpDNA) was prepared in different ratios of FuGENE-HD transfection reagent (volume) to DNA (1μg) as 2/1, 3/1, 4/1 and 5/1 and added to 10^5^ HeLa cells in 6 well plates. Next, we kept fixed volume of FuGENE-HD (4 μl) but HeLa cells cultured in different cell densities including 10^5^, 3×10^5^ and 7×10^5^ cells per well in 6 well-plates 24 hours before transfection time. The transfected cells were kept in 5% CO_2_ at 37˚C for 24 hours. Beta-Galactosidase activity was then determined according to Kit protocol. Moreover, cell viability was measured by trypan blue staining (Invitrogen, USA).

To optimize the transfection into HeLa cells by Lipofectamine^TM^2000, HeLa cells were seeded 16 hours before transfection onto a 6-well plate at four cell densities of 104, 2×10^4^, 5×10^4^ and 10^5^ in 2 ml of DMEM complete medium. The cationic lipid-DNA complexes were prepared by adding different amounts of Lipofectamine^TM^2000 reagent including 4 μl and 5 μl to 1μg of pDNA into polystyrene tubes. Next, after incubating for 20-30 minutes at room temperature, they were added to each well that included serum free media (400 μl). The cells were then incubated at 37˚C for 24 hours prior to gal staining experiments. After 24 hours the rate of cell toxicity was also measured by trypan blue staining. Then, to optimize cell number on transfection time we kept the fixed cell density at 10^5^ per well with 5 μl of Lipofectamine^TM^2000.

In another optimization experiment, in presence of cationic lipid X-tremeGENE, 10^5^ HeLa cells were plated in 6 well-platesin serum supplemented DMEM for 16 hours before transfection. Next the different ratios of lipid XtremeGENE (μl) to DNA (μg) including 4/1 and 5/1 were used. Subsequently, the rate of transfection efficiency and cell toxicity was measured for each condition by beta-gal staining and trypan blue, respectively. To determine cell toxicity, trypan blue staining was used as described before (17). Count of dead cells was carried out under microscope (Nikon, Japan) by hemocytometer. As mentioned above, this experiment was performed for each transfection condition. In this work, all experiments were replicated three times, independently. Also all results were reported as mean along with standard deviation.

Generally, to optimize FuGENE-HD mediated gene delivery into HeLa cells, different cell densities and various volumes of FuGENE-HD were tested. The best transfection rate was conferred up to 43.66 ± 1.52% by using 4 μl of Fu- GENE-HD with 10^5^ cells in each well. Moreover, the majority of HeLa cells, more than 95%, were viable in presence of 4 μl of FuGENE-HD reagent. Also, there was no increase in the rate of gene delivery into HeLa cells at 5 μl of Fu- GENE-HD, despite viability of more than 85% of cells (Fig 1).

**Fig 1:**
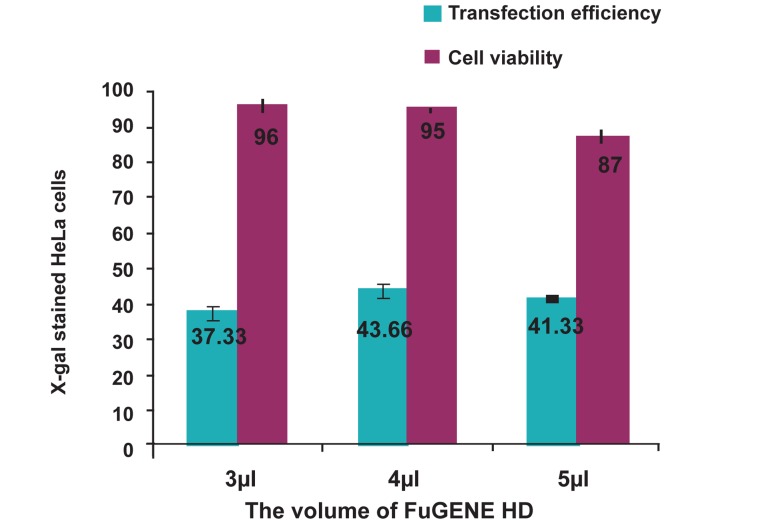
The best result for transfection was achieved in presence of 4 μl FuGENE-HD. Also, increasing the volume of FuGENE-HD, not only did not lead to increase of transfection efficiency, but it also caused more cell toxicity.

Although the rate of transfection into HeLa cells in presence of Lipofectamine^TM^2000 investigated in different condition such as various cell densities and amount of Lipofectamine^TM^2000, HeLa cells were transfected at the average of 31.66 ± 2.5% by using 4 μl of LpiofectamineTM2000 with 10^5^ of HeLa cells per well (Fig 2). In addition, the rate of gene delivery in presence of 5 μl of LpiofectamineTM2000 decreased because of exacerabating cell toxicity.

**Fig 2:**
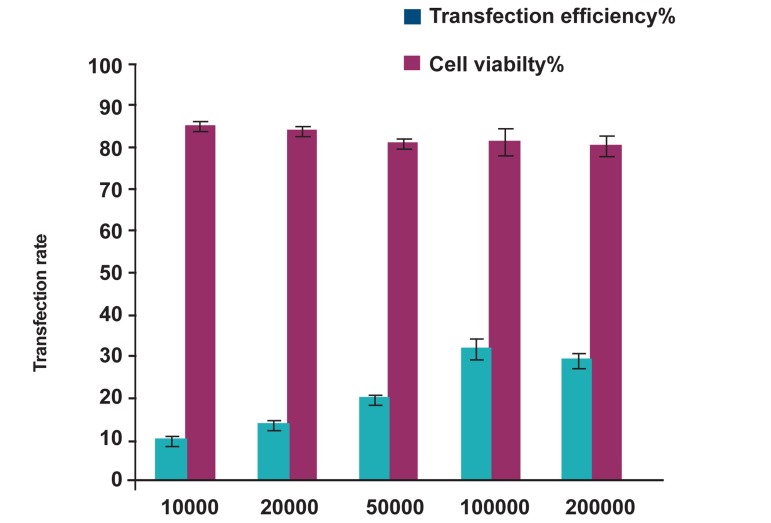
The graph shows optimizing transfection efficiency in different cell densities while the volume of Lipofectamine^TM^2000 fixed at 4 μl.

Further, transfection results with X-tremeGENE showed that it was too toxic for HeLa cells even in less concentration of X-tremeGENE (4 μl) despite being recommended for adherent cells.

In conclusion, the transfection efficiency for HeLa cells in presence of lipid cationic reagents such as Lipofectamine^TM^2000 and X-tremeGENE was less compared with FuGENE-HD as it has been shown in figures 3 and 4.

**Fig 3:**
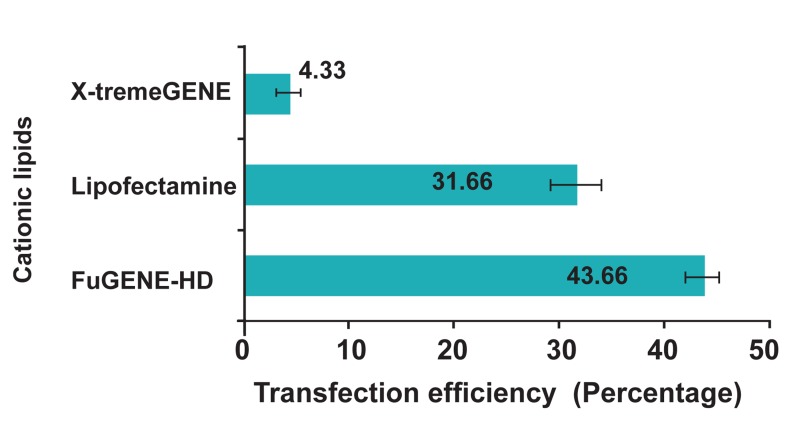
In this comparative graph, the percentage of transfection in presence different cationic lipid reagents has been shown. The rate of transfection of HeLa cells in presence of FuGENE-HD, Lipofectamine^TM^2000 and X-trem- GENE was 43.66 ± 1.52 %, 31.66 ± 2.5% and 4.33 ± 1.15%, respectively.

**Fig 4:**
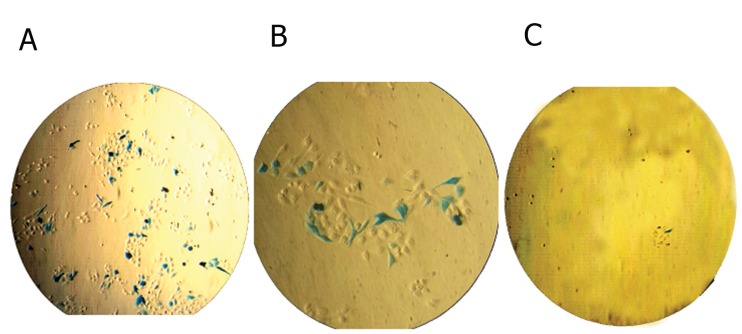
These pictures show the condition of cell viability and the level of transfection efficiency into transfected HeLa cells under microscope in presence of A. FuGENE-HD, B. Lipofectamine^TM^2000 and C. X-tremGENE. The blue cells express beta galactosidase activity.

Having an acceptable gene delivery level into eukaryotic cells is important to determine gene function, protein production from recombinant genes and gene regulation studies (4). For transferring DNA molecules different methods such as viral and non-viral ways exist (5), but there are few data that have been published on FuGENEHD based on transient expression in human cell line such as HeLa cells. Although FuGENE-HD is not common as much as other chemical reagents like Lipofectamine^TM^2000 or X-tremeGENE, HeLa cells could be transfected by FuGENE-HD at a suitable rate (43.66 ± 1.52%) in comparison with Lipofectamine^TM^2000 and X-tremGene (Fig 3). Wiesenhofer and Humpel have showed that the optimal transfection efficiency with FuGENEHD in C6 glioma cells and in primary glial cells was 16.3 ± 0.3% and 5.1 ± 0.37% of total cells, respectively (17). Another report has showed that the best rate for transient transfection of human astrocytoma cell line 1321N1 is achieved by 2.75 μl of FuGENE-HD in combination with 0.5 μl of GFP plasmid (18).

According to our findings, FuGENE-HD based gene delivery into HeLa cells gave reproducible results and detectable expression. Taken together, the significant factors that can influence gene transfection including cell conditions such as cell number and amount of transfection reagents and the type of cell line should be considered for gene delivery experiments, as reported before and shown here (4,16-17).

Our experiments show that, not only transferring reporter gene into HeLa cells by using FuGENE HD was considerable, but also measuring the level of gene transfer were much easier in comparison with Lipofectamine^TM^2000 and X-tremeGENE based transfection because of its low toxicity.

We focused on lipid based gene delivery because it is completely understandable that lipid reagents are suitable and attractive for gene transfer due to more safety and versatility for mammalian cells and it is possible they can be used for *in vivo* trials (13). It makes researchers eager to make new lipid components including DOTAP/DOPE, DCChol/ DOPE and DDAB/DOPE (13). In this context, having enough information for optimizing gene delivery is absolutely necessary such as cell density on the day of transfection, charge of lipids and the presence of serum and antibiotics (17,19). In this work, the conditions recommended such as cell density and ratio of lipids were considered and transfection experiments were carried out in absence of serum and antibiotics.

However, our finding pointed that to achieve the highest gene delivery into HeLa cell line, one cannot rely on the transfection reagents suggested by manufaturers. As a matter of fact, reagents such as Lipofectamine^TM^2000 or X-tremeGENE despite being highly recommended by manufacturers for gene transfer, they were not efficient as much as the reagent FuGENE-HD for HeLa cell line.

Finally, it can be suggested that FuGENE-HD is an appropriate reagent to transfect HeLa cells and the rate of expression of a candidate gene can be evaluated easily after transfection by using this reagent. This reagent is an appropriate candidate instead of other common reagents such as Lipofectamine^TM^2000 or X-tremeGENE which were shown to be toxic.
